# The main criteria for mate selection: The nursing students' perspective in the south‐west of Iran during 2020–2021: A cross‐sectional study

**DOI:** 10.1002/hsr2.949

**Published:** 2022-11-29

**Authors:** Ghazanfar Rafiee

**Affiliations:** ^1^ Department of Medical‐Surgical Nursing Shiraz University of Medical Sciences Shiraz Iran

**Keywords:** mate‐selection criteria, nursing students' perspective, wedding

## Abstract

**Background and Aims:**

Marriage has been a functional and moral relationship between men and women, which leads to the creation of a family and also can result in the birth of a child. This study aimed at determining the mate‐selection criteria of nursing students.

**Methods:**

298 nursing students were considered in this cross‐sectional, descriptive‐analytical study. The study data were collected using a researcher‐made questionnaire with confirmed validity and reliability.

**Results:**

The male and female undergraduate students' mean age was 20–37 and 20–30 years, and male and female graduate students were aged 22–33 and 23–39 years, respectively. From the girls' perspective, 24 out of the 46 criteria were essential for mate selection. From the boys' perspective, 17 criteria were important and five were very low important. The results revealed a significant difference between both genders regarding the importance of 29 criteria. Boys believed that having housekeeping skills, coming from similar cities, and having physical attractiveness were of utmost importance.

**Conclusion:**

Girls paid more attention to psychological and economic criteria in comparison with men. For girls, the husband's nobility and ability to manage the family and for men being a housewife were more important. Despite the differences in some points of view between both genders about considering the criteria for choosing a future spouse, according to all participants, it was important for the participants to include psychological, cultural, familial, and social criteria for choosing a mate selection. It is recommended that, before marriage, the youth should consider the above‐mentioned criteria to choose their future spouse. In addition, as much as possible, boys should have a decent job and an independent income before marriage.

## 1 INTRODUCTION

The need to establish a lasting, intimate, and loving relationship is one of the most important reasons that ultimately causes every man and woman to choose a spouse and husband. Marriage is one of the best human needs to which the holy religion of Islam has given great importance and has considered it as one of the best religious traditions which is a source of innumerable blessings. According to the Quran, marriage is a source of peace for the human soul, which is one of the signs of God's mercy and greatness. The Messenger of God, Peace Be Upon Him, also has said: No building has been established in Islam that is more honorable to God than marriage. Marriage is an act that creates a bond between the two opposite sexes based on lasting sexual relationships.^1^, ^2^, ^3^ Choosing a spouse is one of the most important choices that everyone faces at least once in his/her life. In every choice, a human, willingly or unwillingly, uses criteria as the basis of his/her choice. Some of these criteria are individual and some are common among almost all members of a group, culture, or community. At first glance, having different criteria for choosing a spouse makes this process difficult, but considering the basic criteria makes it easier. A study of the criteria for spouse selection concluded that two categories were included in choosing the right spouse. The first category is the primary criteria (characteristics, ethics, and individual beliefs) that are more stable, and the second category is the secondary criteria (appearance, economic status, and well‐being) that are less tolerable and resilient.^4^, ^5^ In comparison to Western societies, Iranian families play an important role in spouse selection and it is concluded that Iranian families consider selection as the parent's right for their children. There is a premise that individuals bring factors from their families into marriage life that may affect their relationship. Therefore, the choice of a spouse among students should be carefully considered.^6^, ^7^ Due to changes in new values, beliefs, and convictions, young people cannot choose their spouse unseen and unknown and be hopeful for intellectual, value, and normative similarity in their future common life. Thoughts, beliefs, and opinions of the educated persons in spouse selection, as a partner, have a decisive and fundamental role in their future. Spouse selection based on the right criteria is one of the most important factors influencing a successful marriage and one of the worries in society, especially for people who are about to get married, is spouse selection. The purpose of this study was to determine the criteria for spouse selection from the nursing students' perspective.

## 2 METHODS

This descriptive‐analytical, cross‐sectional study was conducted in the southwest of Iran.

### 2.1 Study population

The study population consisted of nursing students. By availability sampling, 298 students (178 girls and 120 boys) in the third and fourth years of bachelor's degree (128 girls and 100 boys) and the last year of the master's degree (50 girls and 20 boys) entered the study. The students who did not complete the questionnaire, who were already married, and who were gay or lesbian were excluded.

### 2.2 Data collection

A researcher‐made electronic questionnaire containing basic criteria in spouse selection was used. As to the validity of the prepared questionnaire, it was distributed among 15 experts; before gathering the data, the reliability of the questionnaire was determined by piloting it on 30 students randomly; its coefficient of internal consistency was measured using Cronbach's alpha (0.92). First, the questionnaires were coded, and at the time of distributing the questionnaires, each participant was given a code, and based on the codes given to the questionnaires and the written consent forms, the coded documents were sent to the participants via the Internet for completion. In case, the participants were willing to participate in the research and complete the questionnaire, a check mark was put by the participant in the relevant space; when the questionnaire was completed by the participants, they returned to the researcher within the specified time. Inclusion criteria were boys and girls in the third and fourth years of nursing undergraduate and final year of graduate program who were willing to participate in the study and signed the consent form. The first part of the questionnaire included the participant's basic demographic questions, including age, sex,‌ place of residence, ‌educational level, number of children per family, number of births, father's education and occupation, mother's education and occupation, parent's monthly income, and their views on cohabitation (Table 1). The second part of the questionnaire included 46 items of spouses' basic selection criteria, based on the Likert scale (Table 2), from “very low important (1), partially important (2), desirable (3), important (4), and essential” (5). The questionnaire items included psychological domain 39% (18/46), economic domain 11% (5/46), social domain 9% (4/46), familial domain 7% (3/46), cultural domain 22% (10/46), and physical domain 11% (5/46) (Table 2). The questionnaire was sent to the participants through virtual space.

**Table 1:** Demographic characteristics of the subjects

	Girls		Boys	
	Bachelor	Master	Bachelor	Master
Father education	29% Diploma, 27% Bs	40% Diploma	32% Diploma, 21% Bs	36% Under Diploma
Father job	44% of all subjects clerk, 35% free job		Unknown	Unknown
Mother education	38% of all subjects under Diploma, 34% Diploma		Unknown	Unknown
Mother Job	82% Housewife, 12% Teacher		Unknown	Unknown

**Table 2:** Frequent distribution of the subjects' attitudes in preference to spouse selection criteria

Domain	Criterion	The priority rate of choosing girls	The priority rate of choosing boys	Chi‐square	*p*‐Value
Psychological	Having the right talent	*64% important	43% desirable	12.32	0.015
	Having emotional control	70% essential	51% important	39.14	0.006
	Having the ability to express emotions	70% essential	59% important	32.17	0.002
	Honesty and truthfulness	61% essential	39% essential	8.109	*p* > 0.05
	Good‐humored	62% essential	37% essential	9.24	*p* > 0.05
	Not being vindictive and stubborn	58% essential	42% essential	2.056	*p* > 0.05
	Be kind	59% essential	41% essential	8.49	*p* > 0.05
	Having intimacy (talkative and warm‐hearted)	59% essential	46% essential	8.48	*p* > 0.055
	He was responsible and accountable	71% essential	65% important	40.11	*p* < 0.0001
	Having decency and modesty	62% essential	38% essential	3.75	*p* > 0.05
	Having chastity (not having a previous relationship)	51% essential	49% essential	3.79	*p* > 0.05
	Be diligent and have high goals	68% essential	45% impotant	18.29	0.001
	Lack of dependent personality	82% essential	56% important	7.53	*p* < 0.0001
	Having the ability to manage problems	78% essential	57% important	7.43	*p* < 0.0001
	Having mental health (not having obsessive‐compulsive disorder, depression, etc.)	64% essential	36% essential	12.38	0.015
	Long suitable period of acquaintance before marriage (6 months to 2 years)	62% essential	38% essential	2.67	*p* > 0.05
	Not addicted to cigarettes, alcohol, drugs, and psychotropic drugs	63% essential	36% essential	22.52	*p* < 0.0001
Economic	Having the right job	81% essential	74% desirable	69.2	*p* < 0.0001
	Have a decent income	83% essential	57% desirable	61	*p* < 0.0001
	Having housing	78% desirable	86% very low important	52.9	*p* < 0.0001
	Having other amenities such as a car	65% desirable	87% very low important	47.9	*p* < 0.0001
	Good financial situation and wealth	67% desirable	78% very low important	32.9	*p* < 0.0001
Social	Having a social status	65% important	54% desirable	27.26	*p* < 0.0001
	Being interested in interacting with others (helping charities and helping people in crisis	60% desirable	40% desirable	42.15	0.004
	Participation	61% important	47% important	1.11	0.025
	Having the social appropriateness of families	64% important	36% important	8.56	*p* > 0.05
	Having the ability to make decisions and communicate socially in critical situations	77% essential	53% important	7.32	*p* < 0.0001
Familial	Having previous family originality and honor	68% essential	45% important	5.67	*p* > 0.05
	Having family and life management	66% essential	51% important	55.15	0.001
	Possessing housekeeping skills	67% desirable	70% important	55.15	0.001
Cultural	Being a fellow citizen or a provincial	58% very low important	67% partially important	9.97	0.041
	Having education appropriateness	66% important	34% important	14.78	0.005
	Having the families cultural appropriateness	62% important	38% important	46.10	0.033
	Having commitment to religious and religious standards (such as praying, fasting, visiting the Imams)	38% desirable	47% very low important	6.76	*p* > 0.05
	Having a moral and religious appropriateness	70% important	55% desirable	19.073	0.001
	Respect each other's rights	64% essential	36% essential	18.63	0.001
	Having an interest in parenting (childbearing)	59% important	41% important	2.47	*p* > 0.05
	Prioritize the opinion of family's fathered	60% desirable	40% desirable	3.54	*p* > 0.05
	Having commitment to family	65% essential	32% essential	10.11	0.039
	Amount of Dowry	63% desirable	37% desirable	4.87	*p* > 0.05
	Amount of Tocher	64% desirable	34% desirable	6.92	*p* > 0.05
Physical	Not having a considerable age difference	60% desirable	40% very low important	9.32	*p* < 0.0001
	Having proper physical attractiveness and beauty	70% desirable	62% important	16.74	0.002
	Having genetic counseling and no inherited diseases in marriage	60% essential	40% essential	18.13	0.01
	No history of disease	64% important	36% important	8.57	*p* > 0.05
	Having physical health (not having a specific disease)	59% essential	41% essential	4.39	*p* > 0.05

* The reported percentage is associated with the categories in which the highest percentage has reported.

### 2.3 Statistical analysis

After gathering the data, they were analyzed using SPSS (Statistical Package for the Social Sciences), version 21. Descriptive statistics methods and frequency distribution tables were used for describing the characteristics of the subjects, also to determine the frequency distribution of the subjects' attitudes in preference to spouse selection criteria, we used two‐sided Chi‐square test through SPSS software version 21 at an error level of > 0.05.

### 2.4 Ethical consideration

All procedures performed in this study for gathering data were in accordance with the ethical standards of the Shiraz University Research Ethics Board with the code of IR.SUMS.REC.1399.1201 and the 1964 Helsinki Declaration and its later amendments or comparable ethical standards.

## 3 RESULTS

In this study, 228 undergraduate (42.5% girls and 33.5% boys) and 70 graduate (17% girls and 7% boys) students were considered. The mean age of undergraduate girls was 22.7 years (age range 20–30), the mean age of undergraduate boys was 22.1 years (age range 20–37), the mean age of graduate girls was 27.2 years (age range 23–39), and the mean age of graduate boys was 26.9 years (age range 22–33). The rest of the demographic information is listed in Table 1. As to 84.5% of the participants (89% of female and 80% of male), cohabitation was a commitment, not a limitation, and there was no significant difference (*p* < 0.05) between the boys' and girls' views in this regard. Girls considered 24 criteria of their future spouse as necessary, but boys reported 16 criteria as important and five criteria as very important (Table 2). Boys and girls did not differ significantly in the preference of importance given to 16 criteria. Chi‐square statistical results showed that there was a significant relationship between gender and 29 criteria for spouse selection (*p* < 0.05) so that, in most criteria, boys and girls in the ratio of the importance of the criteria were different from each other. Besides the criteria of having a housewife skill, boys considered such criteria as residing in the same province and having an attractive physical appearance and appropriate physical beauty to be more important. Gender was effective in responding to these criteria and a significant difference was observed (*p* < 0.05).

## 4 DISCUSSION

For spouse selection, there is a great emphasis on the moral, behavioral, cultural, economic, and religious standards of the future spouse. Girls considered the following psychological criteria (emotional control, ability to express emotions, honesty and truthfulness, good manner, no resentment and stubbornness, kindness, intimacy, responsibility and accountability, decency and modesty, chastity, independent personality, ability to manage problems, mental health, prolonged acquaintance before marriage, no addiction to cigarettes, alcohol and psychotropic drugs) as essential in their future spouse. However, boys considered criteria (emotional control, ability to express emotion, responsibility and accountability, independent personality, and ability to manage problems) as important. There was no significant difference between the criteria that both genders considered essential, and it seems that these criteria are a priority for girls, similar to boys. Shahhosseini et al. showed that psychological factors (good morals, honesty, loyalty, self‐respect, responsibility, and commitment) were the most important criteria for spouse selection.^8^, ^9^ Zaheri et al.^10^ also have shown that this criterion is one of the five main criteria of marriage; girls, in comparison with boys, pay attention a little more to it. From the participant's perspective, the criterion of having a mental health (no obsessive‐compulsive disorder, depression, etc.) is essential, and the observed significant difference between the two genders means that girls pay more attention to this criterion. Carmen et al.^11^ also found that mental health was the most important criterion in spouse selection. According to girls, having a suitable job and a decent income is essential for the future spouse, and having a house, a car, good financial conditions, and wealth are desirable. There was a significant difference between both genders. Because girls need family security and independence to start a family and live together, it is important for them not to have financial problems in their future life, even if they have grown up in a family with no suitable or favorable economic conditions. Abbaszadeh et al.^12^ also mentioned that having a job for the husband was more important for girls than having a wife's job for boys. Khallad^13^ also showed that women in comparison with men cared more about their husband's economic situation. For girls, having a family background and dignity, family and life management with their future spouse were considered essential; boys also considered these criteria important. Boys considered having female housekeeping skills as important, while girls found it desirable. Accordingly, women's housekeeping is more important for boys than girls. Carmen et al.^11^ also mentioned that, besides being a citizen, the first priority in spouse selection is nobility, honesty, and sociability. In our traditional society, housekeeping skills are among the characteristics that are usually specific to women. Also, men pay more attention to such characteristics for spouse selection.^8^, ^14^ The results showed that social criteria were also important. Girls considered having social status, participation, and appropriateness of the families important; inversely, boys considered the same criteria as desirable and important respectively, for their future spouses. It looks that the importance of social domain is somewhat lower for boys than girls, and the participants reported that communication with others (helping charities and people in crisis) were desirable criteria; girls considered the ability to make a decision and social relationship in critical situations as essential, whereas boys considered it as important. Carmen et al.^11^ also stated that sociability was one of the important factors in spouse selection.

From the participant's perspective, having the same educational level (cultural domain) as the couple was important. Both genders agreed on the appropriateness and similarity of the couple's education. However, there was a statistically significant difference between the two sexes, and girls paid more attention to the educational suitability of their future husbands. Walaa et al.^2^ pointed out that the lack of a good intellectual level resulting from education faces men and women with an inner resentment and disturbs their inner peace of mind. Thus, due to the different ways of couples thinking and worldviews and to some extent due to their educational differences, they cannot meet cultural and social expectations of each other; this can affect the couple's interaction and increase the distance between them. Villani et al.^15^ showed that 58.8% of women want their husbands' education level to be higher than their own, but only 6.8% of men were in favor of their spouse's higher education. Participants considered the criteria of respecting each other's rights, having a commitment to the family of their future spouse, as essential and the cultural appropriateness of families, and prioritizing the opinion of the family's fathers as important and desirable respectively. Also, girls stated that having moral and religious appropriateness and a commitment to religious and religious standards (praying, fasting, and visiting the Imams) were important and desirable respectively, but being a fellow citizen or residing in the same province was very low important. As viewed by them, the fact that boys were considered partially important; the boys also believed that having moral and religious appropriateness and commitment to religion and religious standards were of very low importance. It seems that, for boys, it is important that their future spouse is from an urban and provincial area. Apart from having a commitment to religious standards and prioritizing the opinion of family's father, there was a statistically significant difference between the importance of the criteria in both genders. It appears that girls and boys care equally about prioritizing the family's father. It also seems that the observance of religious standards for the boy's future spouses are not as important as the other criteria, the fact that is questionable. Shahhosseini et al.^9^ stated that adherence to religious beliefs, as one of the important cultural‐religious factors, was an important criterion and a factor of stability, consolidation and security in family life and totally in the world. Darban et al.^14^ mentioned that perhaps the females' interest in their spouse's religion is due to the fact that they may think religious people show more commitment to life. From the participants' views, bride dowry and tocher was considered not very important to them as compared to most of the cultural and economic criteria that were essential and important for them. It seems that for the participants these two criteria which were traditionally believed and important for getting married in their predecessors are not a priority for them. Abbaszadeh et al.^12^ showed that as the level of education increases, material stuff is less important for people. The amount of bride dowry and tocher for girls is less important as the level of education increases, while this criterion for boys does not follow a specific trend.

Having good physical attractiveness and beauty were desirable for girls and important for boys. The results show that the beauty of the spouse is one of the most important criteria for boys and to some extent for girls. Borten^4^ also states the reason for its importance for males might be the fact that men can maximize their investment by selecting a wife who is healthy and young.^9^, ^11^ According to the theory of sexual choice in spouse selection, the perception of attractiveness increases the likelihood of acceptance. Although the beauty of the spouse, in our country religious and cultural teaching is considered a criterion, perhaps the excessive attention of today's youth to this criterion is due to the influence of the Western media and the consequent decline of moralities in society. Having genetic counseling and the absence of inherited diseases were essential from the boys' and girls' the perspective; also, girls paid more attention to them. Bijari et al.^1^ showed that the majority of young girls attached great importance to genetic counseling and the absence of inherited diseases in the couple. Having less age difference during the marriage was considered desirable by girls and very low important by boys. It seems that boys prefer the age difference between boys and girls during the marriage to be higher than girls, while for girls, having a lower age difference is desirable; there was a statistically significant difference between the importance of the mentioned criteria in both sexes. From the participants' perspective, it is essential to have physical health (not having a specific disease); there was no significant difference between the two genders and it seems that they care about having physical health. Gholamaliee et al.^16^ have shown that the husband's physical health is very important to the participants.^14^

## 5 LIMITATIONS

Because the study was conducted in the era of Covid‐19 and it was done for the first time through virtual communication, the researcher was faced with problems in preparing and sending the electronic questionnaire to the participants; on the other hand, because the participants were far from the Internet line system and due to lack of having access to the Internet, some participants refused to complete the questionnaire online. This study also was conducted on nursing students in a single faculty and may not reflect the results in the entire country.

## 6 IMPLICATIONS OF THE STUDY

As determined in this study, the main criteria for choosing a spouse would be of use for the students of medical sciences, especially other nursing students who are planning to marry. Students from other disciplines will also be able to get information about the preferences of choosing a spouse adopted by nursing students and compare their views with those obtained in the present study. The findings of this study would also help psychologists and marriage counselors to gain more knowledge about the criteria of educated boys and girls in choosing a spouse and help to improve the satisfactory marital and family relationships of people in the society.

## 7 CONCLUSION

Compared to boys, girls paid more attention to psychological and economic parameters. For girls, the husband's nobility and ability to manage the family and for boys being a housewife were more important. Girls were more concerned with the couples' proximity in terms of education than were men.

In addition, as much as possible, boys should have a decent job and an independent income before marriage. The amount of the wife's dowry and tocher was not very important for both genders. For boys, the physical attractiveness and beauty of their future spouse were important, and, for both genders, doing genetic counseling and not having inherited diseases were essential; also, boys were more concerned about more age differences between the two genders. Despite the differences in some points of view between both genders about considering the criteria for choosing a future spouse, according to all participants, it was important for the participants to include psychological, cultural, familial, and social criteria for choosing a mate‐selection. It is recommended that, before marriage, the youth should consider the above‐mentioned criteria to choose their future spouse.

## AUTHOR CONTRIBUTIONS

**Ghazanfar Rafiee**: Conceptualization; data curation; formal analysis; investigation; methodology; project administration; writing – original draft; writing – review & editing.

## CONFLICT OF INTEREST

The author declares no conflict of interest.

## TRANSPARENCY STATEMENT

The lead author Ghazanfar Rafiee affirms that this manuscript is an honest, accurate, and transparent account of the study being reported; that no important aspects of the study have been omitted; and that any discrepancies from the study as planned (and, if relevant, registered) have been explained.

## ETHICAL CONSIDERATION

All procedures performed in this study for gathering data, were in accordance with the ethical standards of the Shiraz University Research Ethics Board with the code of IR.SUMS.REC.1399.1201 and the 1964 Helsinki Declaration and its later amendments or comparable ethical standards.

## Data Availability

The author confirms that the data supporting the findings of this study are available within the article and its supplementary materials.
